# MiR-221 promotes stemness of breast cancer cells by targeting DNMT3b

**DOI:** 10.18632/oncotarget.5979

**Published:** 2015-10-19

**Authors:** Giuseppina Roscigno, Cristina Quintavalle, Elvira Donnarumma, Ilaria Puoti, Angel Diaz-Lagares, Margherita Iaboni, Danilo Fiore, Valentina Russo, Matilde Todaro, Giulia Romano, Renato Thomas, Giuseppina Cortino, Miriam Gaggianesi, Manel Esteller, Carlo M. Croce, Gerolama Condorelli

**Affiliations:** ^1^ Department of Molecular Medicine and Medical Biotechnology, “Federico II” University of Naples, Naples, Italy; ^2^ IEOS-CNR, Naples, Italy; ^3^ IRCCS-SDN, Naples, Italy; ^4^ Epigenetic and Cancer Biology Program (PEBC) IDIBELL, Hospital Duran I Reynals, Barcelona, Spain; ^5^ Department of Surgical and Oncological Sciences, Cellular and Molecular Pathophysiology Laboratory, University of Palermo, Palermo, Italy; ^6^ Department of Molecular Virology, Immunology and Medical Genetics, Human Cancer Genetics Program, Comprehensive Cancer Center, The Ohio State University, Columbus, OH, USA; ^7^ Department of Surgical and Oncology, Clinica Mediterranea, Naples, Italy

**Keywords:** microRNAs, breast cancer, cancer stem cells, DNMT

## Abstract

Cancer stem cells (CSCs) are a small part of the heterogeneous tumor cell population possessing self-renewal and multilineage differentiation potential as well as a great ability to sustain tumorigenesis. The molecular pathways underlying CSC phenotype are not yet well characterized. MicroRNAs (miRs) are small noncoding RNAs that play a powerful role in biological processes. Early studies have linked miRs to the control of self-renewal and differentiation in normal and cancer stem cells. We aimed to study the functional role of miRs in human breast cancer stem cells (BCSCs), also named mammospheres. We found that miR-221 was upregulated in BCSCs compared to their differentiated counterpart. Similarly, mammospheres from T47D cells had an increased level of miR-221 compared to differentiated cells. Transfection of miR-221 in T47D cells increased the number of mammospheres and the expression of stem cell markers. Among miR-221's targets, we identified DNMT3b. Furthermore, in BCSCs we found that DNMT3b repressed the expression of various stemness genes, such as *Nanog* and *Oct 3/4*, acting on the methylation of their promoters, partially reverting the effect of miR-221 on stemness. We hypothesize that miR-221 contributes to breast cancer tumorigenicity by regulating stemness, at least in part through the control of DNMT3b expression.

## INTRODUCTION

Over the last years, evidence has accumulated on a small subclass of cancer cells with tumorigenic potential and stemness properties [1]. These so-called cancer stem cells (CSCs) have been isolated from a variety of tumor types, including those of the breast [2]. CSCs have two important characteristics: self-renewal and multipotency. These properties make CSCs able to generate new CSCs and simultaneously to produce differentiated mature cells responsible for the cellular heterogeneity of the tumor. CSCs are now considered the driving force of the tumor. In fact, they are the only cells able to regenerate a new tumor when xenografted in to mice, even when only very few cells are injected [2]. Furthermore, CSCs are resistant to conventional chemotherapy and are considered responsible for tumor recurrence [3]. Breast cancer stem cells (BCSCs) are characterized by high CD44 and low CD24 expression, and can be identified as cells able to grow in suspension as spherical structures called mammospheres. Mammospheres derived from tissue specimens survive in non-adherent conditions and differentiate along different mammary epithelial lineages [4]. Within a tumor, CSC enrichment correlates with the grade of the tumor [5].

MicroRNAs (miRs) belong to the non-coding RNA family. They have a size ranging from 20 to 25 nucleotides, and function as endogenous regulators of gene expression. MiRs impair mRNA translation or negatively regulate mRNA stability by recognizing complementary target sites in their 3′ untranslated region (UTR). MiRs are involved in the regulation of many physiological processes, including development, proliferation, and apoptosis, as well as of pathological processes such as cancerogenesis. In breast cancer, miR-21, -155, -96, and -182 have been identified as oncogenes [6–9], whereas miR-125, -205, and -206 have been identified as tumor suppressors [10–12]. MiRs play an essential role also in self-renewal of CSCs. For instance, miR-100 inhibited the maintenance and expansion of CSCs in basal-like breast cancer, and its ectopic expression enhanced BCSC differentiation, controlling the balance between self-renewal and differentiation [13].

In the present study, we investigated whether other miRs are involved in the regulation of stemness in breast cancer. To this end, we isolated BCSCs from patients and analyzed their miR expression profile. We found that miR-221 was significantly up-regulated in BCSCs and was involved in stemness phenotyping through post-transcriptional regulation of DNMT3b, a methyltransferase involved in epigenetic regulation of gene expression.

## RESULTS

### MiRs involved in stemness

**Figure 1:**
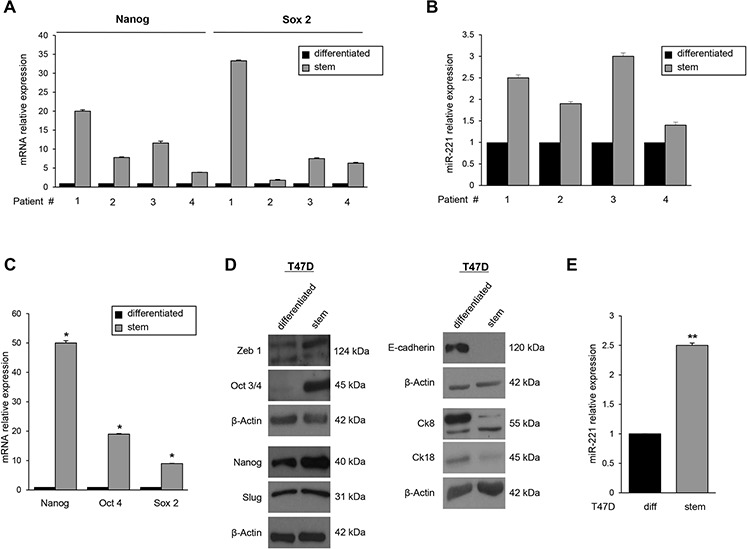
MiR-221 expression in BCSCs and in T47D cell mammospheres **A.** qRT-PCR validated the increase of stem markers *Nanog* and *Sox 2* and **B.** of miR-221 in BCSCs. **C, D.** Nanog, Oct 3/4, Sox 2, Zeb 1, cytokeratin (Ck) 8 and cytokeratin (Ck) 18 were analyzed by qRT-PCR and Western blot and were found up-regulated in T47D stem cells compared to the differentiated counterpart. **E.** qRT-PCR revealed the upregulation of miR-221 in T47D mammospheres with respect to differentiated T47D cells. In C and E, data are mean values ± SD of three independent experiments. Significance was calculated using Student's *t*-test. *, *p* < 0.05; **, *p* < 0.01. Western blots are from representative experiments.

To identify miRs differentially expressed in BCSCs and involved in stemness maintenance, we performed a microarray analysis. The array was performed analyzing the miR expression profile of BCSCs, collected from three patients, compared to that of breast cancer cells growing in adherence (differentiated cells). BCSCs obtained by biopsy digestion were characterized by real time PCR for the expression of the stem cells markers *Nanog* and *Sox2* (Figure 1A) and by their ability to give rise tumors when injected into the flank of nude mice at low number (Supplementary Table S1). The microarray analysis revealed that there was a significant upregulation of miR-221, miR-24, and miR-29a in BCSCs and a down-regulation of miR-216a, miR-25, and let-7d compared to differentiated cells (Table 1). We focused our attention on miR-221, since its role in tumorigenesis has already been reported in several tumor types [14–16]. Microarray results for miR-221 were validated by real time PCR on the same samples and in one additional patient (patient #4) (Figure 1B).

**Table 1:** MiR expression in breast cancer stem cells

Unique ID	Parametric *p*-value	Fold Change (stem *vs* diff)
hsa-miR-221	0.013	1.8
hsa-miR-24	0.003	2.4
hsa-miR-29a	0.012	1.4
hsa-miR-216a	0.004	2.5
hsa-miR-25	0.042	2.2
let-7d	0.034	1.3

Up- and down-regulation of miRs in breast cancer stem cells *vs* differentiated cells. miR screening was performed in triplicate. A two-tailed, two sample *t*-test was used (*p* < 0.05). Four miRs were found significantly upregulated in breast cancer stem cells, and three were found downregulated.

### T47D mammospheres are enriched in stem progenitors and expresses high levels of miR-221

We then studied *in vitro* enrichment and propagation of mammary stem cells with the T47D breast cancer cell line. 1 × 10^4^ T47D cells were grown in DMEM-F12 supplemented with EGF, b-FGF, and B27. After 7 days of culture, we evaluated the stemness markers through real-time PCR and Western blot analysis, and the differentiation markers only through Western blot analysis. The stemness markers Nanog, Oct 3/4, Slug, and Zeb 1 were found upregulated in the suspension cultures, whereas the differentiation markers E-Cadherin, cytokeratin 18, and cytokeratin 8 were upregulated in adherence cultures (Figure 1C and 1D). Moreover, miR-221 expression was increased in T47D mammospheres compared to differentiated cells (Figure 1E), highlighting the correlation of this miR with the stem cell state. Similar results were obtained in additional breast cancer cell lines (MCF-7, MDA-MB-231, and BT-549) (Supplementary Figure S1).

### MiR-221 and stemness phenotype

**Figure 2:**
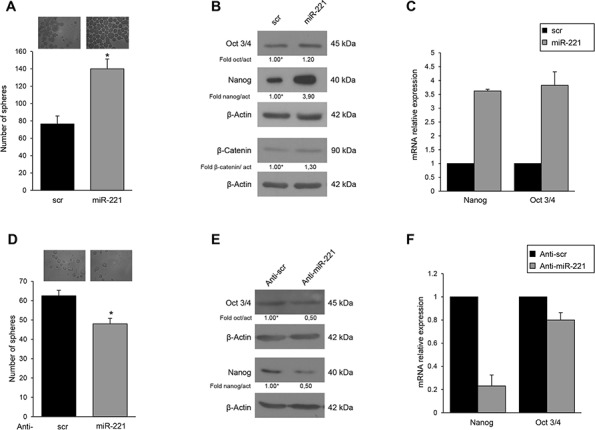
MiR-221 effects on mammospheres and stemness genes expression **A.** T47D cells were transfected with a pre-miR, and mammospheres counted after 6 days. miR-221 induced an increase in the number of mammospheres (140 ± SD versus 76 ± SD). **B, C.** Western blot and qRT-PCR showing that pre-miR-221 transfected in T47D cells upregulates stem cell marker expression. Anti-miR-221 transfection induced a reduction of mammospheres (48 ± SD versus 60 ± SD) **D.** and of stem cell markers **E, F.** Western blots are representative experiments. Data are mean values ± SD of three independent experiments. In A, D, significance was calculated using Student's *t*-test. *, *p* < 0.05.

**Figure 3:**
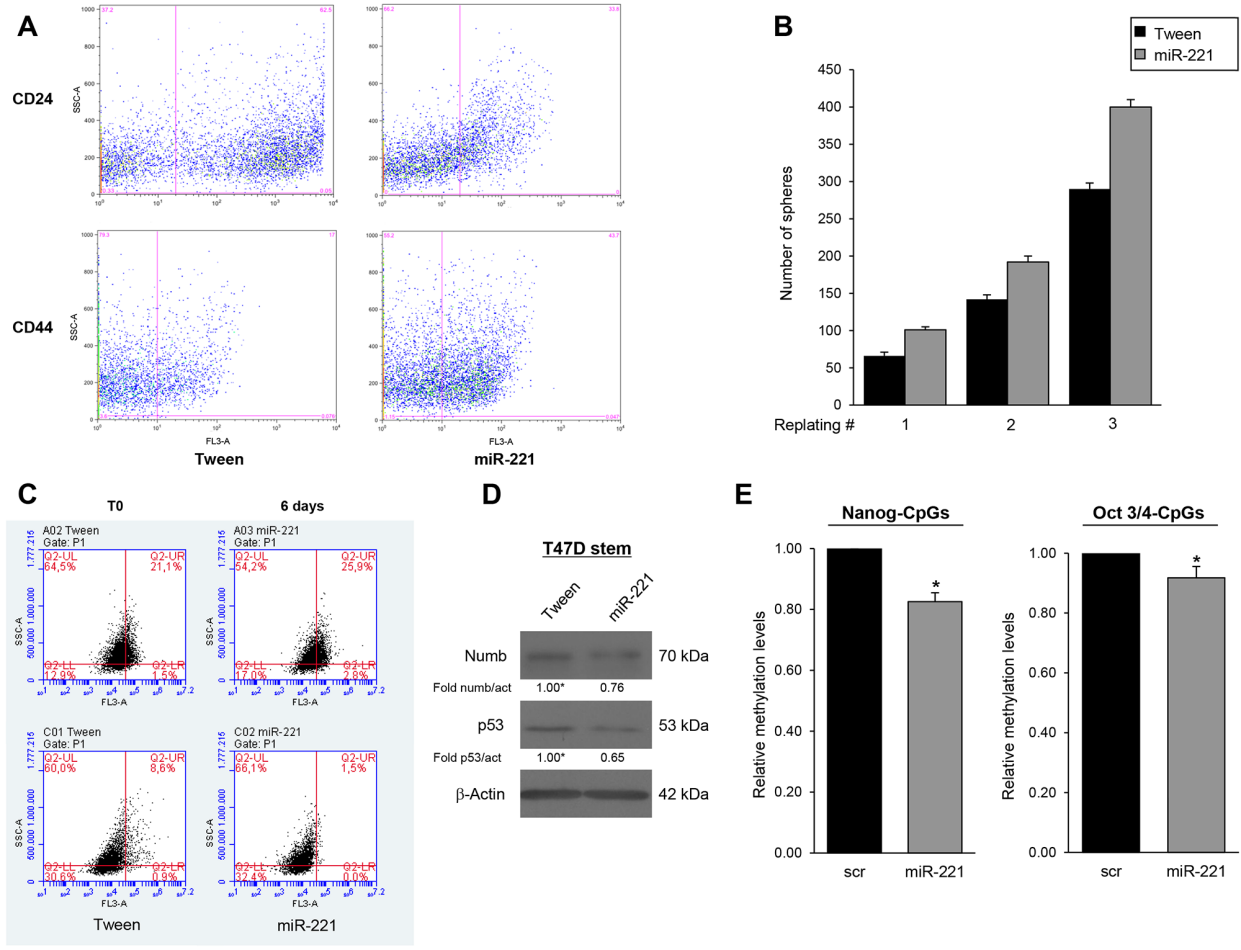
MiR-221 overexpression regulates stemness properties in BCSCs **A.** FACS analysis of CD24/CD44 expression in T47D cells infected with miR-221 lentivirus and control Tween virus (Tw). MiR-221 stable expression induced an increase of CD44 (17% versus 43.7%) and a decrease of CD24 (62.5% versus 33.8%). **B.** Effect of lentivirally mediated overexpression of miR-221 on mammosphere number at the first plating and after dissociation and replating. The data represent the mean value ± SD of two independent experiments. **C.** T47D mammospheres stably infected with the empty vector or miR-221 were evaluated by FACS for PKH26 staining. miR-221 infection induced a decrease of PKH26 in cells (8.6% versus 1.5%). The staining of the two populations was verified at day 0 or after 6 days, as indicated in C. **D.** Asymmetric division was evaluated by Western blotting for Numb and p53. Western blots is representative experiment. **E.** Analysis of methylation change of two consecutive CpGs of *Nanog* and 3 CpGs of *Oct 3/4* promoters (17% and 8% respectively). Methylation values: mean of consecutive CpGs. Significance was calculated using U-Mann Whitney test. *, *p* < 0.05.

To analyze the biological role of miR-221 for the stem cell phenotype, we overexpressed miR-221 in differentiated T47D cells and analyzed different stem cells markers. In order to obtain mammospheres, the cells were kept in stem medium for 6 days. We found that, compared to control, miR-221 overexpression induced a significant increase in the number of mammospheres (Figure 2A) and expression of stem cells markers Nanog, Oct 3/4, and β-Catenin (Figure 2B, 2C). Expression of anti-miR-221 induced an opposite effect (Figure 2D, 2E, 2F). Similar results were obtained in the MCF-7 cell line (Supplementary Figure S2). To further investigate the effect of miR-221 on stem cell properties, we transduced T47D cells with a lentiviral construct encoding miR-221. These stably overexpressing miR-221 cells showed enrichment of the CD44^+^/CD24^−^ population thanks to an increase of CD44 (17% versus 43.7%) and to a decrease of CD24 (62.5% versus 33.8%), as assessed by FACS analysis (Figure 3A). The stable expression of miR-221 in T47D cells induced also an increase in mammosphere number. This ability was enhanced after the first and second replanting, suggesting an expansion of the stem cell compartment (Figure 3B). The increase in sphere number and the upregulation of stemness markers upon miR-221 overexpression indicated an expansion of the stemness pool. In the same manner, the stable expression of miR-221, assessed by qRT-PCR in a breast primary cell line (patient #5), was able to increase sphere formation capacity and Nanog expression also in a primary context (Supplementary Figure S3A, S3B, S3C).

To further verify this phenotype, we assessed the shift from asymmetric to symmetric cell division with PKH26 staining. Fast and symmetrically dividing CSCs tend to rapidly lose PKH26, which then results equally distributed among the daughter cells during each cell division [5, 17]. Mammospheres from T47D cells stably transduced with a Tween control or with miR-221 were labeled with PKH26 and then analyzed by fluorescence microscopy and FACS after 7 days. As shown in Figure 3C, miR-221 overexpression induced a strong decrease of PKH26 (8.6% in Tween cells versus 1.5% in miR-221 cells), suggesting that miR-221 led to an expansion in stem cell number through symmetric division. Asymmetric division was evaluated by the distribution of the cell fate determinant Numb, known to be highly present upon differentiation, and of p53, whose expression is lost in stem cells [17, 18]. Western blotting revealed lower protein expression of both markers in miR-221-overexpressing cells with respect to the Tween control (Figure 3D).

Stemness gene expression is mainly regulated by DNA methylation [19]. For this reason, we decided to evaluate the effect of miR-221 expression on DNA methylation levels of *Nanog* and *Oct3/4* promoters and consequently the regulation of their expression profile. We assessed CpG dinucleotides, which are known to be methylated during differentiation [20, 21]. The 2 CpGs analyzed of *Nanog* promoter region were −83, −36 from to the Transcription Start Site (TSS); whereas the 3 CpGs analyzed of *Oct 3/4* promoter region were +319, +346, +358 from the TSS. Through pyrosequencing analysis, we found that methylation levels at CpGs analyzed on *Nanog* and *Oct 3/4* promoters were significant decreased (17% and 8% respectively) in cells transfected with miR-221 compared to the scrambled control (Figure 3E). Similar results were obtained in additional GpGs analyzed of both promoter regions (10% for CpG at −302, −300, −296 from TSS of *Nanog* and 10% for CpG + 250, +253, +277 of *Oct 3/4*.) (Supplementary Figure S4A).

### MiR-221 specifically represses DNMT3b expression

**Figure 4:**
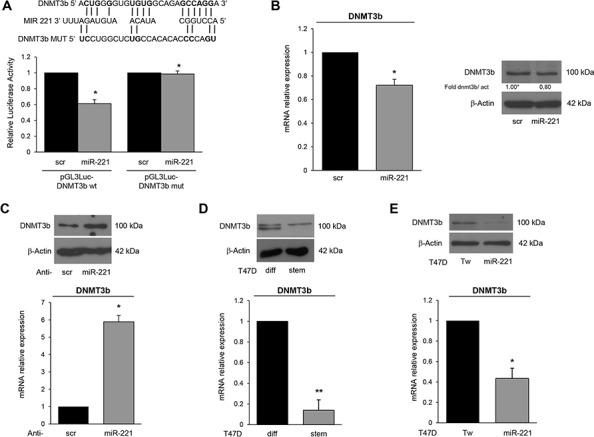
DNMT3b is a direct target of miR-221 **A.** Predicted alignment between the miR-221 sequence and the 3′UTR of *DNMT3b*. Luciferase assay showed that reporter activity was inhibited in T47D cells only in the presence of wild type *DNMT3b* and not with a mutated 3′UTR. The data represent the results of two independent experiments. **B.** MiR-221 transfection downregulated DNMT3b mRNA and protein levels, as assessed by qRT-PCR and Western blotting. **C.** Anti-miR-221 transfection upregulated the levels of DNMT3b. **D.** DNMT3b mRNA and protein were downregulated in T47D stem cells compared to differentiated cells. **E.** DNMT3b mRNA and protein were downregulated in T47D cells stably infected with a miR-221 lentivirus. In B, C, D, E data are mean values ± SD from three independent experiments. Significance was calculated using Student's *t*-test. *,*p* < 0.05; **, *p* < 0.01. Western blot analyses are from representative experiments.

**Figure 5:**
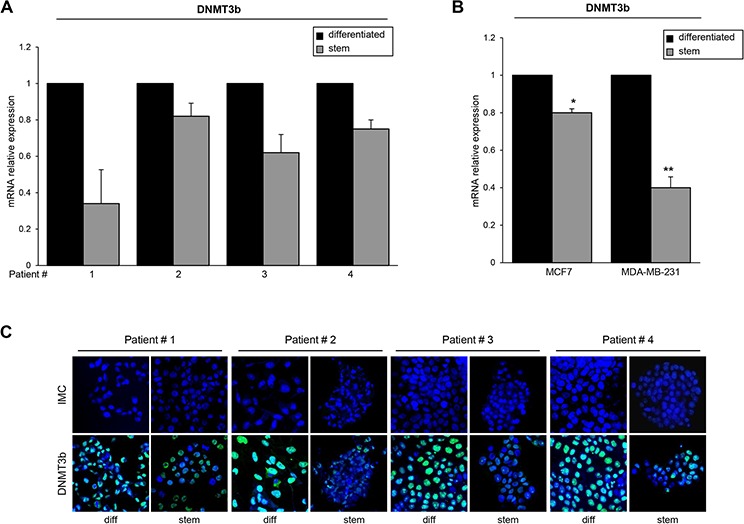
DNMT3b expression in stem and differentiated breast cancer cells **A.** DNMT3b levels were analyzed by qRT-PCR in stem and differentiated breast cancer primary cells or **B.** in MCF7 cells and MDA-231 cells. **C.** Immunofluorecence analysis of DNMT3b expression in stem and differentiated breast cancer primary cells from 4 patients. In B, data are mean values ± SD from three independent experiments. Significance was calculated using Student's *t*-test. *,*p* < 0.05; **,*p* < 0.01.

Thereafter, we investigated miR-221 targets possibly involved in stemness. Among the potential targets predicted by bioinformatics (RNA hybrid-http://www.microRNA.org/, Miranda-http://www.microRNA.org/), we focus our attention on *DNMT3b*, which encodes a DNA methyltransferase involved in de novo DNA methylation [22–24]. To examine whether miR-221 interfered with DNMT3b expression by directly targeting the predicted 3′UTR region, we cloned this region downstream of a luciferase reporter gene in the pGL3 vector. HEK-293 cells are an easy model to use for the luciferase assay thanks to their transfection efficiency. HEK-293 cells were transfected with the reporter plasmid in the presence of a negative control miR (scrambled miR) or miR-221. As shown in Figure 4A, DNMT3b 3′UTR luciferase reporter activity was significantly repressed by the addition of miR-221 compared to the scrambled sequence. This luciferase activity was not affected by miR-221 overexpression in the presence of a mutant construct in which the seed sequence was cloned inversely (Figure 4A). In order to find a causative effect between miR-221 and DNMT3b expression, we transfected T47D cells with a pre-miR-221 for 48 h and then analyzed DNMT3b levels by Western blot and qRT-PCR. We found that DNMT3b protein and mRNA levels were downregulated after miR-221 overexpression (Figure 4B). Similar results were obtained when we transfected miR-222, which shares a similar seed sequence with miR-221 (Supplementary Figure S5). In contrast, anti-miR-221 induced an increase of DNMT3b levels (Figure 4C). Then, we verified DNMT3b expression in stem and differentiated T47D cells. As shown in Figure 4D, DNMT3b expression was lower in stem cells and inversely correlated with miR-221 levels. Furthermore, DNMT3b expression was reduced in T47D cells transfected with miR-221 lentiviral vectors (Figure 4E). We also observed a reduction of DNMT3b levels by qRT-PCR and immunofluorescence in stem cells compared to differentiated primary cells (Figure 5A, 5C), as well as in MCF7 cells and MDA-MB-231 cells (Figure 5B).

### miR-221 controls stemness by inhibiting DNMT3b expression

**Figure 6:**
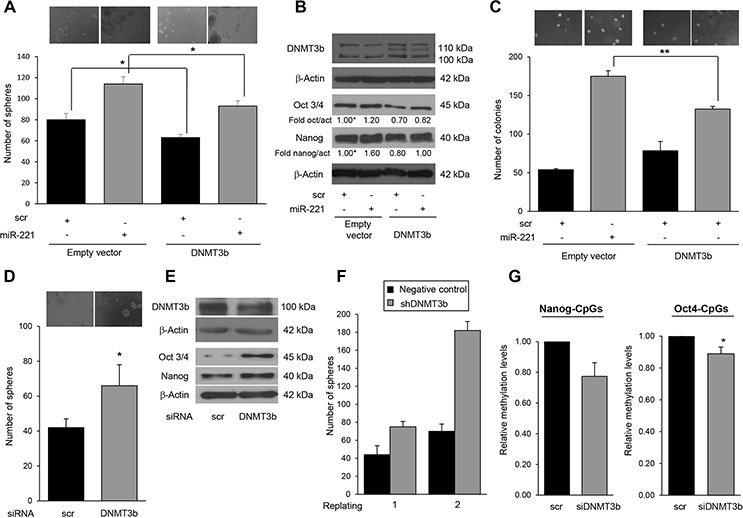
MiR-221 regulates stemness by targeting DNMT3b **A.** DNMT3b represses mammosphere formation, as assessed with a mammosphere counting assay. Data are mean values ± SD from three independent experiments. Significance was calculated using Student's *t*-test. *, *p* < 0.05. **B.** Stem cell markers assessed by Western blotting. **C.** MiR-221 transfection in T47 stem cells induced an increase in the number of colonies, as assessed by a soft agar assay. Co-transfection of DNMT3b and miR-221 rescued this effect. Data are mean values ± SD from two independent experiments. Significance was calculated using Student's *t*-test.**, *p* < 0.01; Transient DNMT3 silencing mimicked the effect of miR-221 on **D.** sphere formation and **E.** stem markers, whereas stable silencing mimicked miR-221 behavior **F.** on sphere number in a plating assay. In D, data are mean values ± SD of three independent experiments. Significance was calculated using Student's *t*-test. *, *p* < 0.05. In **G.** Pyrosequencing analysis of cells transfected with a DNMT3b siRNA showed a significant decrease in methylation levels at CpGs analyzed on *Nanog* and *Oct 3/4* promoters compared to the scrambled control cells (23% and 11% respectively). Significance was calculated using U-Mann Whitney test. *, *p* < 0.05. Western blot analyses are representative experiments.

DNMT3b is a master regulator of Nanog and Oct 3/4 expression and, through its methylation activity, represses their expression during embryogenesis [25]. Therefore, we wondered whether the stemness features observed upon expression of miR-221 were related to DNMT3b downregulation and, consequently, to a reduced methylation activity. We transfected T47D cells with a DNMT3b cDNA and investigated the effect on stem marker expression and mammosphere formation. As shown in Figure 6A–6B DNMT3b inhibited mammosphere formation (63 versus 80) and Nanog, and Oct 3/4 expression. In contrast, treatment with a specific si-DNMT3b-mRNA induced an increase in mammosphere number (Figure 6D) and upregulated Nanog and Oct 3/4 protein levels (Figure 6E). Furthermore, to establish a causal link between miR-221-mediated DNMT3b downregulation and stem cell phenotype, we performed a rescue experiment by transfecting T47D cells simultaneously with pre-miR-221 and a DNMT3b cDNA lacking the 3′UTR. We found that the effect of miR-221 on Nanog and Oct 3/4 expression was abolished by DNMT3b cDNA overexpression (Figure 6B). Its effect on the number of mammospheres (114 versus 93) (Figure 6A) and on growth in soft agar was also partially reverted (Figure 6C). To further asses the role of DNMT3b, we evaluated mammosphere number in T47D cells stably transfected with a shRNA targeting DNMT3b. The expression of a DNMT3b short hairpin increased the number of mammospheres in DNMT3b-silenced T47D cells, an effect enhanced after the first replating (Figure 6F).

We then hypothesized that DNMT3b affects the methylation pattern of *Nanog* and *Oct 3/4* promoter regions, influencing their expression. Pyrosequencing analysis revealed that cells transfected with a DNMT3b siRNA showed a significant decrease (23% and 11%, respectively) in methylation levels at CpGs analyzed on *Nanog* and *Oct 3/4* promoters compared to the scrambled control cells (Figure 6G). Similar results were obtained in additional GpGs analyzed of both promoter regions (20% and 5% respectively) (Supplementary Figure S4B).

## DISCUSSION

Breast cancer is the leading cause of death in woman and is characterized by an elevated heterogeneity, different responses to therapy, and metastatic variability among patients [26]. It represents the first human carcinoma for which a putative cancer stem cell subpopulation has been isolated on the basis of its CD44^+^/CD24^−^/low antigenic phenotype [2]. However, little is known on the mechanisms regulating the ability of BCSCs for self-renewal and to initiate tumors.

Recently, miRs have been found to be critical regulators of several cellular events [27]. By their ability to target hundreds of mRNAs, they can induce a rapid switch in cell fate and a fine exchange in genome expression; they are now accepted as major post-transcriptional regulators. The importance of miRs in gene expression regulation is emphasized by the finding that they are often deregulated in cancer [28]. MiRs may affect cancer development, progression, and response to therapy. Interestingly, some miRs have been reported to regulate CSC phenotype, since their modulation has been shown to contribute to the maintenance or triggering of the phenotype in different cancer models. For instance, it was found that miR-22 induces an expansion of the breast CSC compartment and induces metastasis by downregulating a member of the TET family [29]. Different members of the miR-200 family were found downregulated in CSCs isolated from colorectal, head and neck, prostate, and breast cancer compared to their non-CSC counterparts [30–33]. Expression of miR-200 represses EMT, contributing thus to the progression of cancer by promoting invasion, metastasis [34], and stemness phenotype.

In the present study, we identify miR-221 as an important player in the control of the CSC homeostasis. We provide evidence that miR-221 is expressed at higher levels in the stem cell population of primary and T47D cells compared to differentiated cells. MiR-221 has been found overexpressed in a number of human tumors by us and others [14, 35–38]. The relevance of this miR as an oncogene in breast cancer is reported by several papers, demonstrating the broad spectrum of action miR-221 and its regulation of several features of tumorigenesis [35, 39]. MiR-221 is found often abnormally expressed in breast cancer [40] and recent studies have found that it may be responsible for resistance to tamoxifene [41]. MiR-221 promotes tumorigenesis of triple negative breast cancer through the alteration of key genes of the EMT process, such as E-cadherin, Slug, and Snail [42]; its expression is under the direct control of Slug, suggesting the existence of a miR-221–EMT regulatory loop [43]. In addition, it was reported that miR-221 is upregulated in prolonged mammosphere cultures of MCF7 cells undergoing EMT and with downregulated ER-alpha [44], and that it is able to sustain breast cell hierarchy in normal and malignant breast cells, probably via EMT [45].

Here we demonstrate that miR-221 induces expression of pluripotency-associated genes, such as Nanog, Oct 3/4, and β—Catenin, enforcing stemness and mammosphere formation. miR-221 downregulates DNMT3b expression, modifying BCSC phenotype. DNMT3a, DNMT3b, and DNMT1 are members of the DNA methylation machinery. During DNA replication, DNMT1 recognizes the CpGs present on the parent strand and methylates the corresponding CpG sites of the newly synthesized strand [46, 47]. In contrast, DNMT3a and DNMT3b are responsible for de novo DNA methylation predominantly during early development [48, 49]; in addition, they are important for stable inheritance of some DNA methylation, and the silencing of both enzymes in embryonic stem cells (ESCs) determines a progressive loss of DNA methylation at critical sites of the genome, such as repetitive and single copy elements [50]. Moreover, ESCs lacking both *DNMT3a* and *DNMT3b* progressively lose differentiation potential after several cell passages, but are able to maintain self-renewal [25, 50, 51]. Interestingly, it has been demonstrated that DNMT3a and DNMT3b ablation induces aberrant expression of Nanog and Oct 3/4 in ESCs [52]. The role of DNMT3b in cancer development is not still clear. Although DNMT3b was classically considered an oncogene, due to its role in the hypermethylation of tumor suppressor genes during tumor progression in lung, breast, colon, and bladder cancers [53, 54], several reports also indicate a tumor suppressor behavior at an advanced tumor stage [55, 56]. Therefore, DNMT3b may act as a tumor suppressor or an oncogene depending on tumor stage or on the type of tumor cell population. In the present study, we demonstrate that DNMT3b represses the expression of Nanog and Oct 3/4 and increases the number of breast cancer cell spheres. Thus, DNMT3b downregulation may represent an advantage for cancer development, driving the expansion of the stem cell compartment. Further experiments are necessary to elucidate the mechanism through which DNMT3b regulates stemness, other than acting directly on *Nanog* and *Oct 3/4* promoters.

In conclusion, we have identified a new mechanism by which miR-221 affects the tumor stemness phenotype of breast cancer cells, providing more information on the oncogenic role of miR-221 in breast cancer.

## MATERIALS AND METHODS

### Cell and mammosphere culture

Differentiated breast tumor cells from three patients (#1, #2, #3) and BTSCs (breast tumor stem cells) were obtained as previously described [57] and were used for microRNA array. T47D cells were grown in RPMI 1640 supplemented with 10% heat-inactivated fetal bovine serum (FBS), 2 mM L-glutamine, and 100 U/ml penicillin/streptomycin. For mammosphere culture, single cells were plated at a density of 1,000 cells/ml. Cells were grown in serum-free DMEM-F12 (Sigma, Milan, Italy) supplemented with B27 (Life technologies Milan Italy), 10 ng/ml EGF (Sigma, Milan, Italy), 20 ng/ml βFGF (BD Biosciences, Milan, Italy), and 1X antibiotic–antimycotics (Life technologies, Milan, Italy). After 5–7 days, mammospheres, which appeared as spheres of floating viable cells, were collected by gentle centrifugation (800 rpm) and dissociated with 0.25% trypsin for 5 min. HEK-293 cells were grown in DMEM supplemented with 10% heat-inactivated FBS and 100 U/ml penicillin/streptomycin.

### Cell and sphere transfection

For transient transfection with miRs, cells at 50% confluence were transfected using Oligofectamine (Life Technologies Milan Italy) with 100 nM of pre-miR-221, scrambled, or anti miR-221 (Ambion, Life Technologies Milan Italy). In order to overexpress DNMT3b, cells were transfected with Lipofectamine 2000 and 3 μg of DNMT3b cDNA, a kind gift of Ana Portela (IDIBELL, Barcelona, Spain). To transiently knockdown DNMT3b gene expression, a pool of DNMT3b siRNAs is transfected using Lipopfectamine 2000 at a final concentration of 100 nM (Santa Cruz Biotechnology, MA, USA). To stably knockdown DNMT3b, cells were infected with a shDNMT3b (Santa Cruz Biotechnology, MA, USA) and the expression of DNMT3b studied by qRT-PCR in pooled cell populations (data not shown).

### Transduction with viral vectors

T47D cells and a primary breast cell line obtained from patient #5 were infected using Tween miR-221 or Tween control vector, as already described by Quintavalle et al. [14]. Briefly, on the day of infection, the medium was removed and replaced with viral supernatant with the addition of 4 mg/ml of polybrene (Sigma Aldrich, Milan Italy). Cells were then centrifuged in their plate for 45 min in a Beckman GS-6KR centrifuge, at 1800 rpm and 32°C. After centrifugation, cells were kept overnight in a 5% CO_2_ incubator at 37°C. After exposure, cells were washed twice with cold PBS and fresh medium added. 48 h after the transduction, cells were washed with PBS, harvested with trypsin/EDTA, and analyzed by FACS for GFP expression.

### Protein isolation and western blotting

Cells were washed twice in ice-cold PBS, and lysed in JS buffer (50 mM HEPES pH 7.5 containing 150 mM NaCl, 1% Glycerol, 1% Triton X100, 1.5 mM MgCl_2_, 5 mM EGTA, 1 mM Na_3_VO_4_, and 1X protease inhibitor cocktail). Protein concentration was determined by the Bradford assay (BioRad, Milan, Italy) using bovine serum albumin as the standard, and equal amounts of proteins were analyzed by SDS-PAGE (12.5% acrylamide). Gels were electroblotted onto nitrocellulose membranes (G&E Healthcare, Milan, Italy). Membranes were blocked for 1 h with 5% non-fat dry milk in Tris Buffered Saline (TBS) containing 0.1% Tween-20, and incubated at 4°C overnight with the primary antibody. Detection was performed with peroxidase-conjugated secondary antibodies using an enhanced chemiluminescence system (ThermoEuroclone, Milan, Italy). Primary antibodies used were: anti-Zeb-1, -Oct 3/4, -Nanog, -cytokeratin 18, and -cytokeratin 8 (Santa Cruz Biotechnologies, MA, USA), anti-DNMT3b (Abcam, MA, USA), and anti-β-actin (Sigma Aldrich, Milan, Italy).

### miRNA microarray

5 μg of total RNA from each sample was reverse transcribed using biotin end-labeled random-Octamer oligonucleotide primer. Hybridization of biotin-labeled complementary DNA was performed on Ohio State University custom miRNA microarray chips (OSU_CCC version 3.0), which contain 1150 miR probes, including 326 human and 249 mouse miR genes, spotted in duplicate. The hybridized chips were washed and processed to detect biotin-containing transcripts by streptavidin-Alexa647 conjugate and scanned on an Axon 4000B microarray scanner (Axon Instruments, Sunnyvale, California, USA).

Raw data were normalized and analyzed with GENESPRING 7.2 software (zcomSilicon Genetics, Redwood City, CA, USA). Expression data were median-centered with the GENESPRING normalization option and the global median normalization of the BIOCONDUCTOR package (http://www.bioconductor.org), which produced similar results. Statistical comparisons were done with the GENESPRING ANOVA tool, predictive analysis of microarray (PAM), and the Significance Analysis of Microarray (SAM) software (http://www-stat.stanford.edu/∼tibs/SAM/index.html).

### Mammosphere forming assay

Mammospheres were resuspended in 0.5% agar (Bacto-Agar, Difco Laboratories) and layered on a preformed 0.8% agar layer using 60 mm Petri dishes (BD). Colonies were counted under an inverted microscope (Nikon, Milan, Italy) and then photographed.

### RNA extraction and real-time PCR

Total RNAs (miR and mRNA) were extracted using Trizol (LifeTechnologies, Milan, Italy) according to the manufacturer's protocol. Reverse transcription of total miRNA was performed using miScript reverse Transcription Kit (Qiagen, Milan Italy), for mRNA we used SuperScript^®^ III Reverse Transcriptase (Life Technologies, Milan, Italy). Quantitative analysis of Nanog, Oct 3/4, Sox2, β-Actin (as an internal reference), miR-221, and *RNU6B* (as an internal reference) was performed by real time PCR using specific primers (Qiagen, Milan, Italy), miScript SYBR Green PCR Kit (Qiagen, Milan Italy), and iQ™ SYBR Green Supermix (Bio-Rad, Milan, Italy), respectively. Experiments were carried out in triplicate for each data point, and data analysis was performed with software (Bio-Rad, Milan Italy).

### Luciferase assay

The 3′ UTR of the human DNMT3b gene was PCR-amplified using the following primers: *DNMT3b*-Fw:5′GCTCTAGACAGCCAGGCCCCAAGCCC3′; *DNMT3b*-Rv: 5′GCTCTAGAACCTCAGGCTACCCCTGC3′, and cloned downstream of the Renilla luciferase stop codon in pGL3 control vector (Promega, Milan, Italy). An inverted sequence of the miR-binding sites was used as negative control. HEK-293 cells were co-transfected with 1.2 μg of plasmid and 400 μg of a Renilla luciferase expression construct, pRL-TK (Promega, Milan, Italy), with Lipofectamine 2000 (Life Technologies, Milan, Italy). Cells were harvested 24 h post-transfection and assayed with Dual Luciferase Assay (Promega, Milan, Italy) according to the manufacturer's instructions. Three independent experiments were performed in triplicate.

### DNA methylation analysis by pyrosequencing

Bisulphite conversion of 500 ng of each DNA sample was performed with EZ DNA Methylation-Gold Kit (Zymo Research, Milan Italy) according to the manufacturer's recommendations. PCR for *Nanog* and *Oct 3/4* promoters was performed with 1 μl of bisulphite converted DNA under standard conditions with biotinylated primers using an annealing temperature of 60°C. Primer sequences are given in Supplementary Table S2 and were designed with PyroMark Assay Design 2.0. PCR products were observed on 2% agarose gels before pyrosequencing analysis. Reactions were performed in a PyroMark Q96 System version 2.0.6 (Qiagen, Milan, Italy) and the methylation values of the CpG dinucleotides were obtained using Pyro Q-CpG 1.0.9 (Qiagen, Milan, Italy). The 2 CpGs analyzed of *Nanog* promoter region were −83, −36, from to the Transcription Start Site (TSS); whereas the 3 CpGs analyzed of *Oct3/4* promoter region were +319, +346, +358 from the TSS. Additional GpGs analyzed of both promoter regions were −302, −300, −296 from TSS of *Nanog* and + 250, +253, +277 from TSS of *Oct 3/4*.

### Immunofluorescence

Immunofluorescence was performed on cultured BCSC cytospins fixed with 2% paraformaldehyde for 20 minutes at 37°C, washed and permeabilized with PBS plus 0.1% Triton X-100 for 10 min on ice. After washing, cells were stained overnight at 4°C using antibodies against DNMT3b (Abcam-ab13604) or isotype-matched controls at appropriate dilutions. Then cells were labeled with FITC-conjugated secondary antibodies for 1 h at 37°C. Nucleus counterstaining was performed using Toto-3 iodide. Samples were analyzed by confocal microscope.

### Statistical analysis

All experiments were repeated at least twice. Continuous variables are given as mean ± 1 standard deviation (SD). For two-group comparison, Student's *t*-test was used to determine differences between mean values for normal distribution. All data were analyzed for significance using GraphPadPrism 6 software (San Diego, CA, USA); a probability level <0.05 was considered significant throughout the analysis.

## SUPPLEMENTARY FIGURES AND TABLES


