# Supplementary material for: MiR-221 promotes stemness of breast cancer cells by targeting DNMT3b
Source: Oncotarget. 2015 Oct 19;7(1):580–92. doi: 10.18632/oncotarget.5979 (PMC4808019; doi:10.18632/oncotarget.5979)
Supplement: Supplementary file 1 [file oncotarget-07-0580-s001.pdf]

SUPPLEMENTARY FIGURES AND TABLES

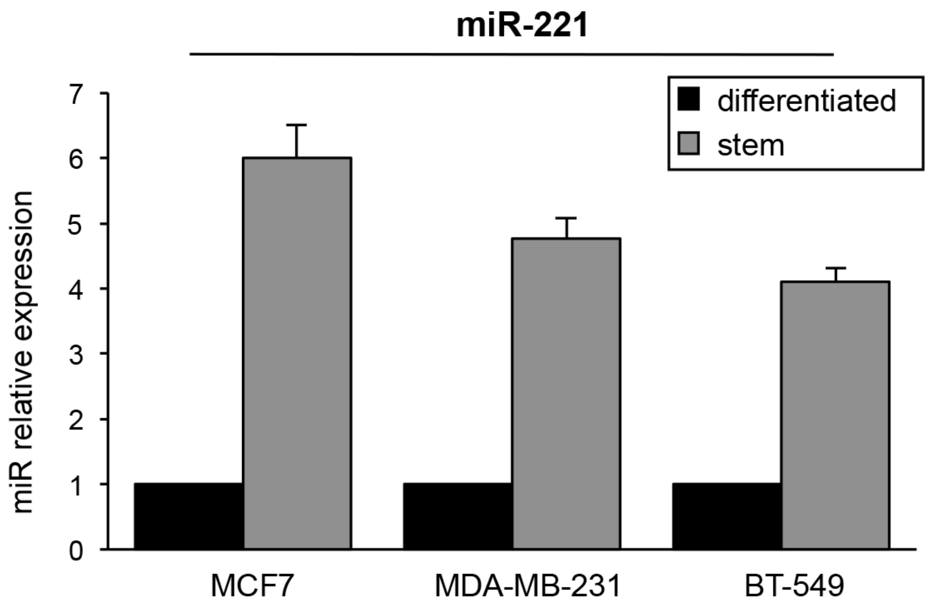

Supplementary Figure S1: MiR-221 expression in MCF7, MDA-MB-231, BT-549 breast cancer cells. qRT-PCR revealed the upregulation of miR-221 in breast mammospheres with respect to differentiated cells. The experiment was repeated twice.

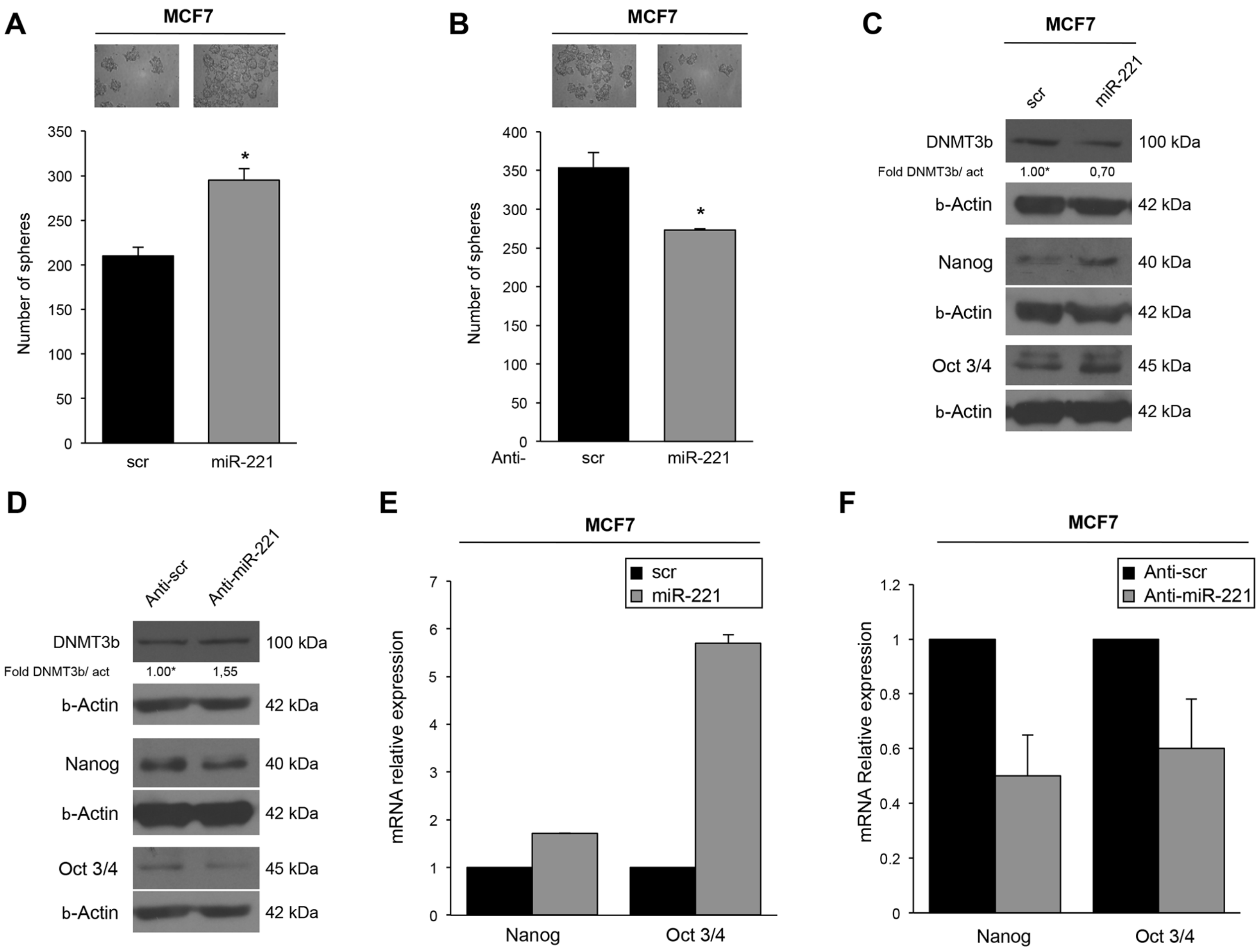

Supplementary Figure S2: Effect of miR-221 expression on stemness properties in MCF7 cells. A, B. MCF7 cells were transfected with a pre-miR or anti-miR, and mammosphere counted after 6 days. MiR-221 induced an increase in the number of mammospheres (295 ± SD versus 210 ± SD), whereas anti-miR-221 transfection induced a reduction of mammospheres (273 ± SD versus 354 ± SD). **C, E.** Western blot and qRT-PCR showing that pre-miR-221 transfected in MCF7 cells upregulates stem cell marker expression. **D, F.** whereas anti-miR-221 has an opposite effect. Western blot analyses are representative experiments. In A and B, Data are mean values ± SD of three independent experiments. Significance was calculated using Student's *t*-test. *, $p < 0.05$.

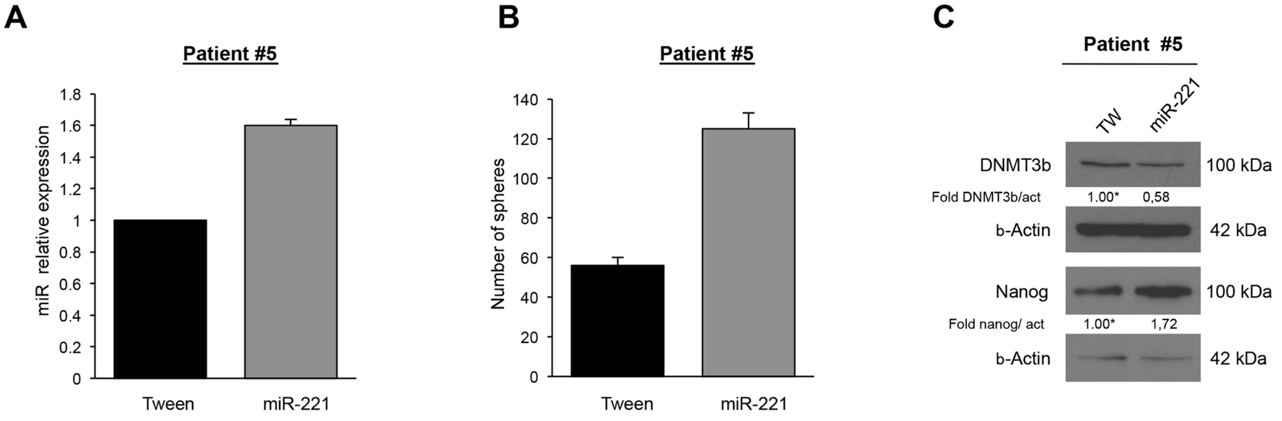

Supplementary Figure S3: Effect of stable expression of miR-221 on stemness in primary cells. A. Sable expression of miR-221 in patient #5 was confirmed by real time PCR. **B.** Stable expression of miR-221 upregulated the number of mammospheres in primary breast cancer cells. **C.** MiR-221 infection induced a decrease of DNMT3 and an increase of Nanog, as assessed by Western blot. The experiments were repeated at least twice.

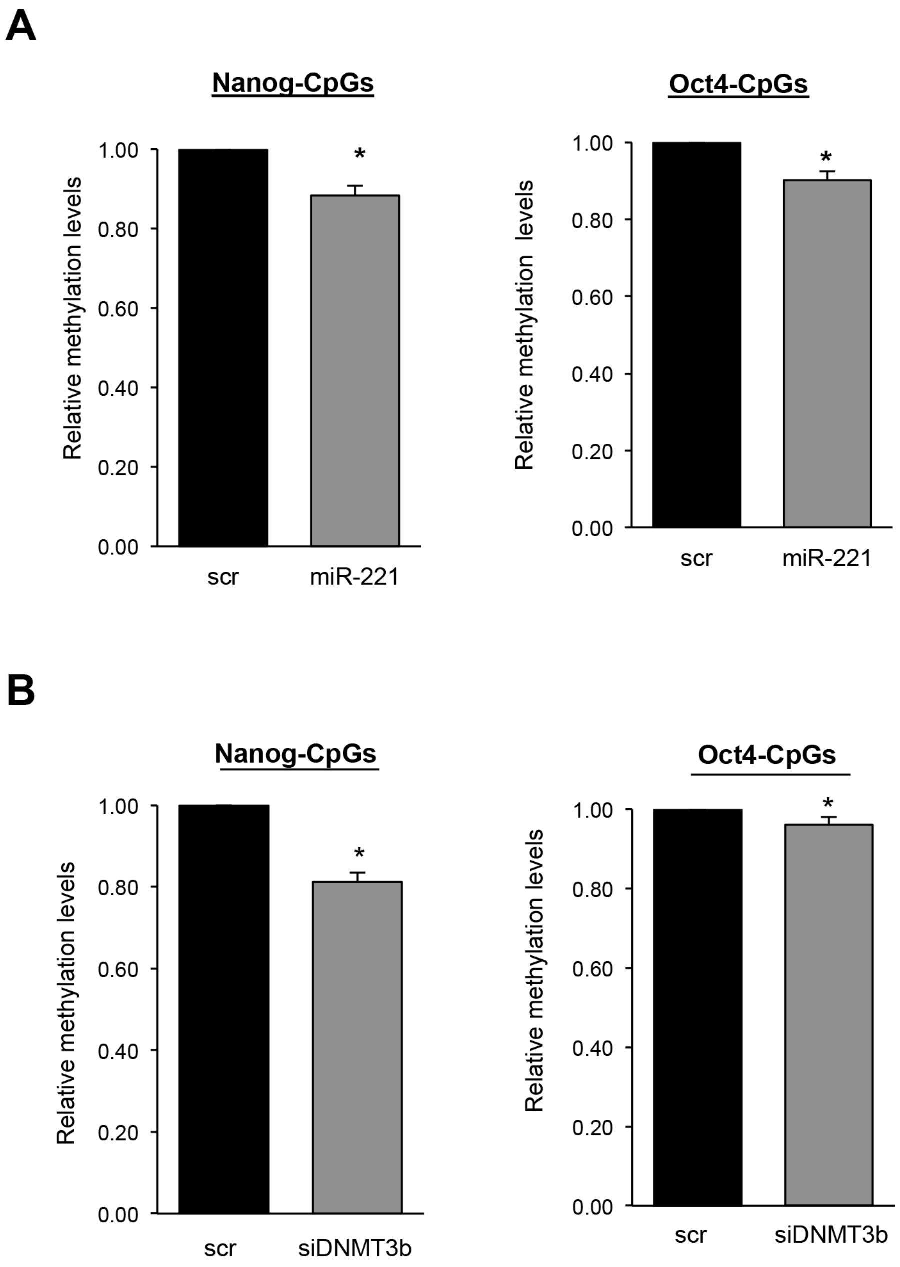

Supplementary Figure S4: Pyrosequencing analysis of CpGs of Nanog and Oct 3/4 promoter regions. A. Analysis of methylation change on CpGs of *Nanog* and *Oct 3/4* promoters after miR-221 expression (10%). **B.** Analysis of methylation change on CpGs of *Nanog* and *Oct 3/4* promoters after DNMT3b siRNA (20% and 5% respectively). Methylation values: mean of consecutive CpGs. Significance was calculated using *U*-Mann Whitney test. *, $p < 0.05$.

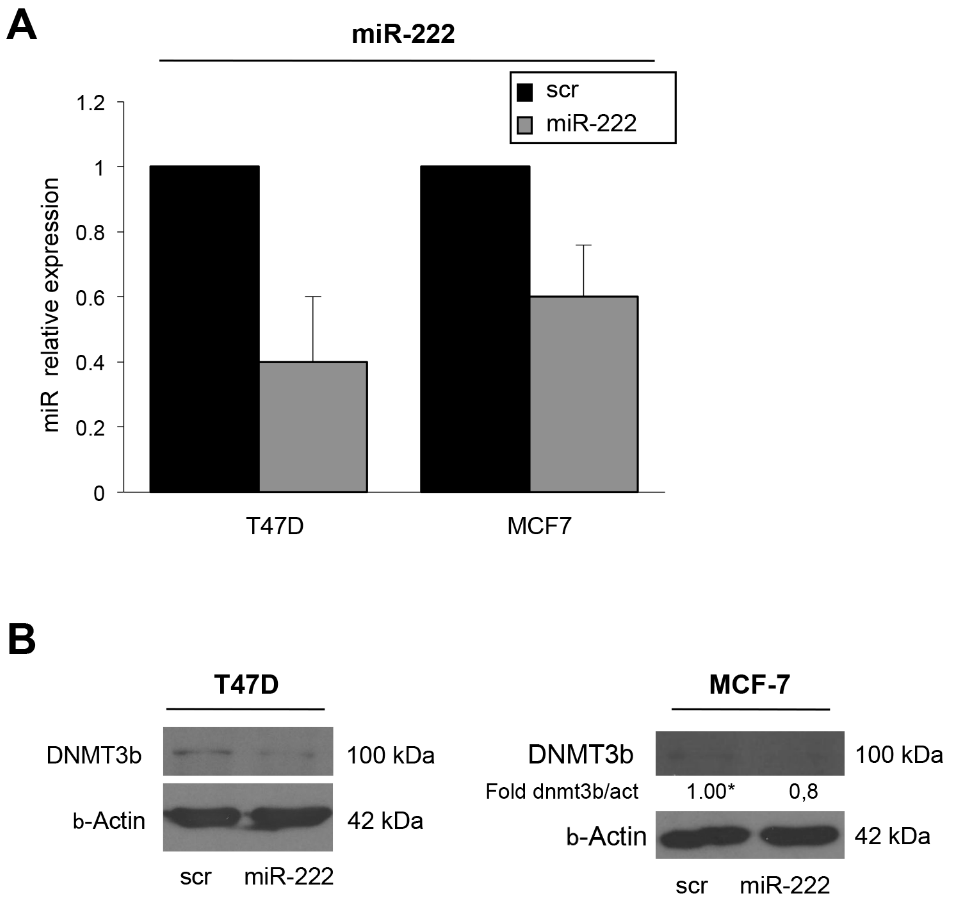

Supplementary Figure S5: Effect of miR-222 on DNMT3b expression. A. miR-222 transfection downregulated DNMT3b mRNA and **B.** protein levels, as assessed by qRT-PCR and Western Blot both in T47D than MCF7. Western blots are from representative experiments.

Supplementary Table S1: Primary breast cancer cells were injected into the flank of nude mice in the indicated number

	Patient	Cell number injected	Xenograft formation
**Spheres**	1-2-3-4	3×10^5	Yes
**Adherent cells**	1-2-3-4	2×10^6	No

Tumor formation was observed after 4 weeks only when sphere cells were injected.

Supplementary Table S2: Primer sequences for pyrosequencing analysis

Pyrosequencing primers	Sequence
Nanog Reverse 1	CCTACATAATAACATAAAACAACCAACTCA
Nanog Forward 1	[Bio]AAGTATTTGTTGTTGGGTTTGTTTTTAGG
Nanog Sequencing 1	TTAAACCCACCCCTC
Nanog Reverse 2	[Bio]AAAATAACTACAAAATAACCCAAACTAAAT
Nanog Forward 2	TTTTTAATTTATTGGGATTATAGGGGTGGG
Nanog Sequencing 2	ATTATAGGGGTGGGT
OCT-4 Reverse 1	[Bio]CCCCATCRAAATTACTCTCCACCC
OCT-4 Forward 1	TTGGGTTAGGTTTTGAGGTGT
OCT-4 Sequencing 1	GGGGGATTTTTTTATGTTT
OCT-4 Reverse 2OCT-4 Forward 2	[Bio]CCCCCCACAAAACTCATACTTAGGTGGTGGAGGTGAT
OCT-4 Sequencing 2	GTGGAGGTGATGGGT
OCT-4 Reverse 3	[Bio]CCATCAAACTACCCTATCATAACC
OCT-4 Forward 3	TGGAGTGGGGTTAGTGTT
OCT-4 Sequencing 3	GGGGTTAGTGTTTTAAG

(1) indicates primers used for the first analysis;
(2–3) indicate primers used for the second analysis.
